# Evaluation of laboratory predictors for intravenous immunoglobulin resistance and coronary artery aneurysm in Kawasaki Disease before and after therapy

**DOI:** 10.1007/s10067-022-06366-x

**Published:** 2022-09-21

**Authors:** Jie Liu, Bingbing Ye, Danyan Su, Suyuan Qin, Weiying Zhao, Yusheng Pang

**Affiliations:** Department of Pediatrics, First Affiliated Hospital, Guangxi Medical University, No 6, Shuangyong Road, Nanning, Guangxi Zhuang Autonomous Region 530000 China

**Keywords:** Before and after treatment, Intravenous immunoglobulin, Kawasaki disease, Risk factor

## Abstract

**Objectives:**

We aimed to evaluate the clinical and laboratory characteristics of patients with Kawasaki disease (KD) before and after therapy.

**Methods:**

Patients with KD were divided into different groups according to their responsiveness to initial intravenous immunoglobulin (IVIG) treatment and coronary status. The clinical and laboratory parameters before and after therapy were compared. Multivariate analysis was performed to identify the independent risk factors, and the receiver operating characteristic (ROC) curve was applied to assess and compare the prediction ability of risk factors and their fluctuations.

**Results:**

Of the 153 patients included in the study, 41 (26.8%) had IVIG resistance and 37 (24.2%) had developed CAA. After stratifying by therapy response, the two groups differed in the levels of total bilirubin (TSB), albumin, and sodium, neutrophil-to-lymphocyte count ratio (NLR), platelet-to-lymphocyte count ratio (PLR), TSB-to-albumin (B/A) ratio, and prognostic nutritional index (PNI) before IVIG, and in the white blood cell count (WBC), neutrophil count, levels of hemoglobin, C-reactive protein (CRP), alanine aminotransferase (ALT), and albumin, NLR, PNI, capillary leakage index (CLI), and systemic immune-inflammation index (SII) after IVIG. Multivariate analysis revealed that the B/A ratio before IVIG and CLI and SII after IVIG were significantly and positively associated with IVIG resistance and that there was a larger decline in the B/A ratio and smaller decline in CLI and SII pre- and post-treatment in the IVIG-resistant group than in the IVIG-responsive group. However, no statistical differences in the fluctuations of the B/A ratio, CLI, and SII as well as all parameters before and after therapy were observed in patients with and without CAA. ROC curve analyses found a greater AUC value of post-treatment parameters (0.751 and 0.706 for CLI and SII, respectively) compared with pre-treatment parameters (0.654 for B/A ratio) in predicting IVIG resistance; however, the predictive ability of the fluctuations in risk factors before and after therapy was not superior to that of baseline values.

**Conclusions:**

The B/A ratio before IVIG and CLI and SII after IVIG were risk factors for IVIG resistance in patients with KD, independent of CAA development.

**Table:** 

**Key Points** *• A high total bilirubin-to-albumin ratio before IVIG and high capillary leakage and systemic immune-inflammation indices after IVIG may indicate an increased risk of intravenous immunoglobulin resistance in patients with Kawasaki disease.* *• Post-treatment parameters were superior to pre-treatment parameters in terms of prediction; therefore, rapid and repeated assessment of risk factors before and after treatment must be considered in children in whom the vital signs and symptoms do not improve after treatment.*

**Supplementary Information:**

The online version contains supplementary material available at 10.1007/s10067-022-06366-x.

## Introduction

Kawasaki disease (KD), which is an acute, self-limiting systemic vasculitis of unknown etiology that affects small- and medium-sized arteries, was first reported by Tomisaku Kawasaki in 1967 [1]. Epidemiological investigations have shown that the incidence of KD, which has been increasing annually, is particularly high in East Asia, especially in Japan, Korea, and China [2–4]. With in-depth research related to KD, there is consensus that patients who are resistant to intravenous immunoglobulin (IVIG) therapy are at high risk of coronary artery aneurysm (CAA) development [5, 6]. Thus, identifying patients who are more likely to be resistant to IVIG treatment and the risk factors for IVIG resistance have become an important research direction in the field of KD, resulting in the development of several scoring systems, such as the Kobayashi, Egami, and Sano [5, 7, 8], for predicting IVIG resistance based on many risk factors. To date, there is no consensus on the risk factors for KD, and there is poor evidence supporting the use of various risk factors or scoring systems for predicting IVIG resistance in non-Japanese populations [9–12].

Studies have discovered that among high-risk patients for initial IVIG resistance, the incidence of adverse coronary artery outcomes has been significantly reduced by adding corticosteroids or cyclosporine to standard-dose IVIG and aspirin in the primary treatment of KD [13, 14]; therefore, studies are necessary to uncover novel parameters in the prediction of IVIG resistance among patients with KD. Given that a single risk factor for IVIG resistance is not necessarily transferable among populations, parameters merging the risk factors, such as the prognostic nutritional index (PNI) and systemic immune-inflammation index (SII), were correlated with increased incidence of IVIG resistance and CAA development and may have better discriminatory ability than any indicator alone, as reported in the past two years [15–20]; nevertheless, these results remain controversial. Moreover, most studies used data acquired before IVIG treatment, and few studies have included laboratory data both before and after treatment and taken into consideration the dynamic changes in these predictors. It is widely demonstrated that systemic inflammation is critical in the onset and progression of KD and that fluctuating inflammatory markers may be used to assess inflammation more precisely as compared to static ones. Studies found that laboratory parameters shortly after IVIG treatment may be more helpful in identifying patients with KD who are at greater risk of CAA development and in guiding subsequent management as compared with those before IVIG infusion. Additionally, the fluctuations in the risk factors might possess greater predictive power for CAA development in patients with KD compared with baseline values [21, 22]. Therefore, the present study focused on evaluating the clinical and laboratory characteristics of patients with KD before and after therapy who were grouped by responsiveness to the initial IVIG treatment and coronary status, and comparing the prognostic accuracy of risk factors with that of their fluctuations pre- and post-treatment.

## Subjects and methods

### Subjects

We retrospectively reviewed the clinical records of children with KD at a single center in China between January 2013 and August 2021. All patients met the standard diagnostic criteria for KD. Positive echocardiogram findings of CAA were defined by a body surface area-adjusted z-score of coronary segments exceeding 2.5 in accordance with AHA criteria [1]. Patients were excluded if they were treated without IVIG or received IVIG treatment after 10 days from fever onset. Patients who received IVIG or steroid therapy in other medical facilities, or those who had incomplete data that were required for statistical analyses, were also excluded.

Upon diagnosis, all patients received initial IVIG (2 g/kg given as a single intravenous infusion) and oral aspirin (30–50 mg/kg per day) until the resolution of fever for at least 72 hours; they then received aspirin (3–5 mg/kg per day) for two months from disease onset. Patients with IVIG resistance, defined as persistent or recrudescent fever (temperature >38.0°C) for at least 36 hours but not longer than 7 days after completion of the initial IVIG infusion, received additional rescue treatment such as a second dose of gamma globulin and steroid (e.g., prednisone or methylprednisolone) therapy. Based on their responsiveness to the initial IVIG treatment and coronary status, all patients were divided into subgroups consisting of the following: the IVIG-responsive (*n* = 112) and IVIG-resistant (*n* = 41) groups and those without CAA (*n* = 116) and with CAA (*n* = 37) groups.

Laboratory data obtained before and 24–36 h after IVIG treatment were collected. Some timing errors of sample collection may exist because of the retrospective nature of this study without controls. Laboratory parameters before IVIG included the white blood cell (WBC) count, neutrophil count, lymphocyte count, hemoglobin concentration, platelet count, and levels of alanine aminotransferase (ALT), aspartate aminotransferase (AST), total serum bilirubin (TSB), serum albumin, sodium, and C-reactive protein (CRP). Laboratory parameters after IVIG treatment included all the above except the serum sodium concentration. We calculated the neutrophil-to-lymphocyte (NLR) count ratio, platelet-to-lymphocyte (PLR) count ratio, TSB-to-albumin (B/A) ratio, and capillary leakage index (CLI, defined as CRP-to-albumin ratio) based on the indicators above. Moreover, the prognostic nutritional index (PNI) was calculated by an equation that includes the serum albumin level and lymphocyte count [PNI = albumin (g/L) + 5 × lymphocyte count (×10^9^/L)]. The systemic immune-inflammation index (SII) was calculated by an equation that included the neutrophil, lymphocyte, and platelet counts [SII = platelet count × (neutrophil count/lymphocyte count)]. Laboratory values before and after IVIG treatment were compared between groups. The fractional change (FC) of variables that showed a significant difference between the groups was also compared. FC was defined as follows: FC = (Y−X)/X, where X and Y represent data before IVIG and 24–36 h after IVIG treatment, respectively. Echocardiography data on the coronary arterial internal diameters of the proximal right coronary artery, left main coronary artery, and left anterior descending artery, which were performed at diagnosis then once a week within one month after disease onset, were obtained. A pediatric cardiologist and echocardiographers determined the diagnosis and measurements of the CAA based on the maximal z-score of all coronary arteries.

### Statistical analysis

Normality of distribution was verified using the Shapiro–Wilk and homogeneity tests. Data with a normal distribution were expressed as mean ± standard deviation, and the two-sample independent t-test was used to compare the data between the groups. Data that did not have a normal distribution were expressed as median (four-digit interval) [*P*_*50*_ *(P*_*25*_*, P*_*75*_*)*] and were compared between the groups using the Mann–Whitney U test. Enumeration data were expressed as percentages (%). The Chi-square or Pearson Chi-square tests were used to perform intergroup comparisons. Variance inflation factors were used to check for collinearity, and significant indices were analyzed using multivariate logistic regression to determine risk factors. The optimum threshold for the significant parameter was constructed using receiver operating characteristic (ROC) curves, and the area under the curve (AUC) was calculated to evaluate the capacity of the risk factors. The *P*-values were two-tailed, with *P* < 0.05 considered statistically significant. Statistical analyses were performed using SPSS, version 26.0 (IBM Corp., Armonk, NY, USA).

## Results

### Baseline characteristics

**Fig. 1:**
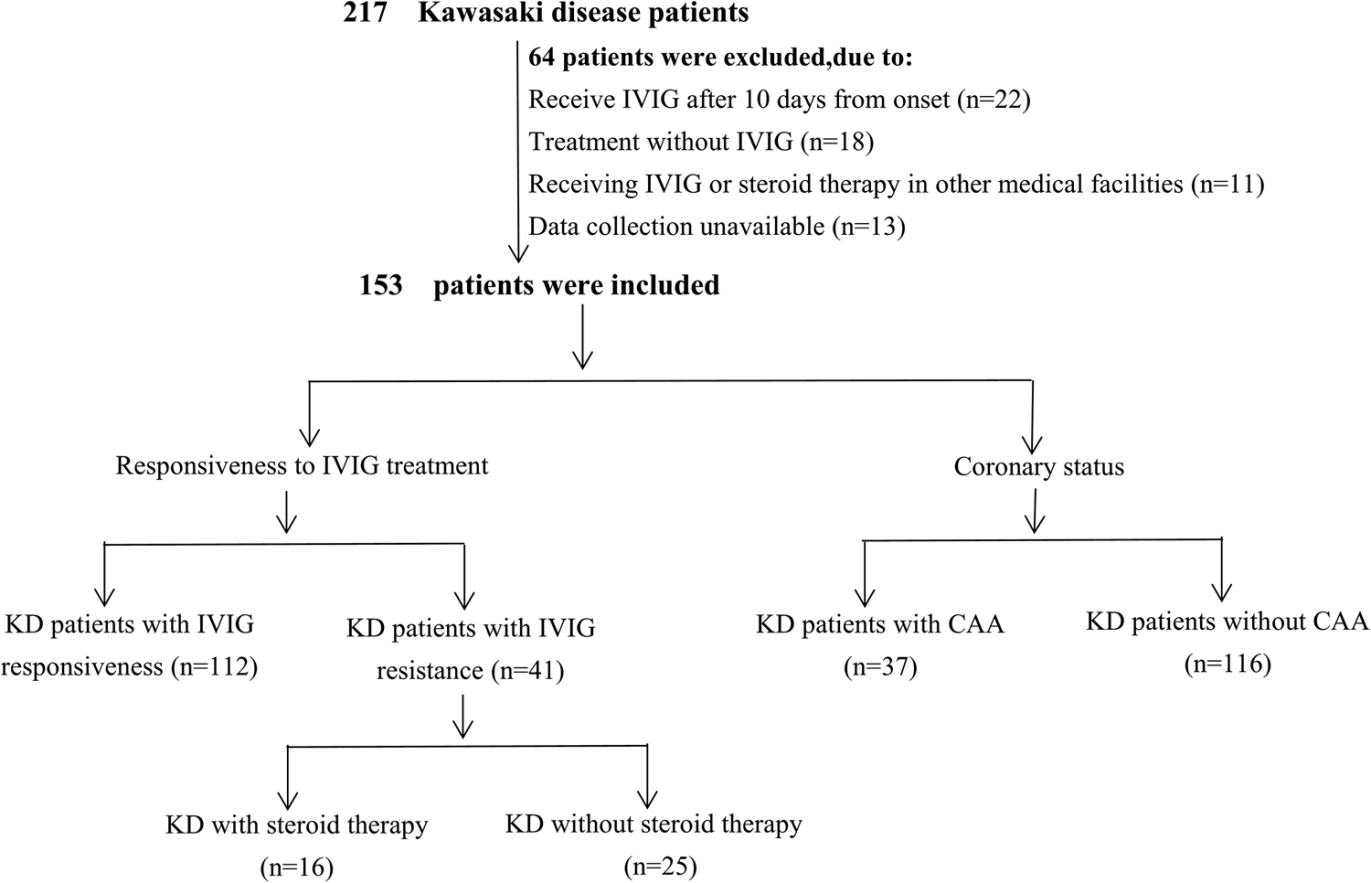
Flow chart of the study. IVIG, intravenous immunoglobulin; KD, Kawasaki disease; CAA, coronary artery aneurysm

A total of 217 children diagnosed with KD were enrolled in this study: 18 were excluded due to treatment without IVIG, 22 due to having received IVIG treatment after 10 days from fever onset, 13 due to incomplete data, and 11 due to having received IVIG or steroid therapy in other medical facilities. Of the 153 patients included, 41 (26.8%) had initial IVIG resistance and 37 (24.2%) had developed CAA. Additionally, 16 (10.5%) had received steroid therapy due to unremitting fever after completion of the second IVIG treatment (Fig. 1). The routine use of antihistamines before IVIG infusion to avoid hypersensitivity reactions is not recommended in our medical center, and no patients discontinued treatment due to infusion reactions. Additionally, no other biological agents, such as infliximab, cyclosporine, anakinra, cyclophosphamide, or plasma exchange, were administered during this period. The patients’ mean age was 30 months (range 3–117 months), with a male-to-female ratio of 2.6:1 (110 boys and 43 girls). There were 29 cases (19.0%) of incomplete KD and no disease recurrences or deaths in either group.

### Comparisons of baseline characteristics

The results of the analysis of the baseline characteristics by the study group are shown in Table 1. In terms of demographic and clinical characteristics, IVIG-resistant patients did not differ from IVIG-responsive patients as well as patients with and without CAA (*P* > 0.05).

**Table 1:** Comparisons of baseline characteristics between patients with Kawasaki disease, by subgroups

	Therapy response			Coronary status		
	IVIG-resistant (n=41)	IVIG-responsive (n=112)	*P*–*value*	CAA (n=37)	NCAA (n=116)	*P*–*value*
Demographic characteristics						
Age [month]	22.00 (13.00, 51.00)	24.00 (14.25, 40.50)	0.547	24.00 (12.50, 40.50)	23.00 (14.25, 43.00)	0.878
< 6 months	2 (4.9)	3 (2.7)	0.869	3 (8.1)	2 (1.7)	0.170
< 12 months	7 (17.1)	19 (17.0)	0.987	8 (21.6)	18 (15.5)	0.389
Male	30 (73.2)	80 (71.4)	0.832	31 (83.8)	79 (68.1)	0.065
BMI [kg/m^2^]	15.34±1.88	15.67±1.55	0.677	15.79±1.80	15.58±1.59	0.492
Clinical characteristics						
Conjunctival injection	30 (73.2)	75 (67.0)	0.464	24 (64.9)	81 (69.8)	0.571
Changes in lips and oral cavity	28 (68.3)	73 (65.2)	0.719	24 (64.9)	77 (66.4)	0.866
Polymorphous exanthem	32 (78.0)	77 (68.8)	0.260	22 (59.5)	87 (75.0)	0.069
Cervical lymphadenopathy	24 (58.5)	56 (50.0)	0.349	21 (56.8)	59 (50.9)	0.532
Changes in extremities	16 (39.0)	39 (34.8)	0.631	11 (29.7)	44 (37.9)	0.365
Incomplete KD	5 (12.2)	24 (21.4)	0.197	5 (13.5)	24 (20.7)	0.332
Fever duration before admission [day]	5.88±2.73	5.78±2.53	0.830	5.97±3.56	5.75±2.19	0.223
Days of illness at primary treatment [day]	6.22±1.35	6.77±1.73	0.089	6.97±1.55	6.51±1.67	0.137
IVIG resistance	-	-		12 (32.4)	29 (25.0)	0.374
CAA	12 (29.3)	25 (22.3)	0.374	-	-	

Data are expressed as mean with standard deviation, median (interquartile range), or number (percentage)

IVIG, intravenous immunoglobulin; CAA, coronary artery aneurysm; NCAA, no coronary artery aneurysm; KD, Kawasaki disease

### Laboratory parameters before and after IVIG treatment between groups

Before IVIG, the IVIG-resistant group had higher NLR, PLR, TSB, and B/A ratio but lower levels of serum sodium, serum albumin, and PNI than the IVIG-responsive group. After IVIG, NLR remained higher, and albumin and PNI remained lower in the IVIG-resistant group. Additionally, the WBC and neutrophil counts, CRP and ALT levels, CLI, and SII were higher, and the levels of hemoglobin were lower, in the IVIG-resistant group than in the IVIG-responsive group. However, statistically significant differences were not found between the groups regarding the lymphocyte count, platelet count, and AST level before and after IVIG. Unexpectedly, all indicators were not statistically different before and after treatment in patients with and without CAA (Table 2).

**Table 2:** Comparisons of pre- and post-IVIG laboratory parameters between patients with Kawasaki disease, by subgroups

	Pre-IVIG		*P*–*value*	Post-IVIG		*P*–*value*	Pre-IVIG		*P*–*value*	Post-IVIG		*P*–*value*
	resistant	responsive		resistant	responsive		CAA	NCAA		CAA	NCAA	
WBC (×10^9^/L)	13.65±7.02	15.51±6.59	0.131	14.60 (10.57, 17.60)	9.19 (7.43, 12.35)	<0.001	16.34±7.77	14.59±6.35	0.169	11.97±5.23	10.79±4.65	0.197
Neutrophils count (×10^9^/L)	9.94±5.25	10.28±5.71	0.739	8.41 (4.71, 12.28)	3.57 (2.13, 5.84)	<0.001	11.58±6.64	9.74±5.14	0.080	6.09±4.40	5.24±3.98	0.276
Lymphocytes count(×10^9^/L)	2.81±2.92	3.73±2.63	0.065	3.85±2.07	4.81±4.33	0.177	3.33±2.44	3.54±2.83	0.688	4.44±1.99	4.58±4.31	0.843
NLR	4.96 (1.96, 10.11)	2.89 (1.80, 5.11)	0.021	1.97 (0.93, 4.92)	0.93 (0.49, 1.43)	<0.001	5.49±4.73	4.40±4.19	0.184	1.22 (0.67, 1.64)	1.02 (0.53, 1.90)	0.415
Hemoglobin (g/L)	104.51±15.30	105.99±14.12	0.574	94.07±11.95	101.88±13.26	0.001	103.82±15.37	106.16±14.11	0.392	98.74±14.36	100.12±13.05	0.585
Platelet count (×10^12^/L)	322.91±144.15	354.61±148.26	0.240	439.27±223.63	473.27±208.62	0.382	337.16±151.76	348.97±146.50	0.673	441.52±187.75	471.37±220.12	0.459
PLR	151.13 (77.38, 333.33)	105.06 (71.43, 166.67)	0.015	107.05 (78.14, 178.36)	107.92 (74.63, 159.77)	0.382	152.89±126.15	141.67±98.66	0.576	109.45 (74.91, 151.14)	106.71 (76.52, 172.90)	0.359
CRP (mg/L)	97.20 (52.78, 147.75)	75.31 (37.27, 136.13)	0.191	60.50 (15.04, 135.84)	14.94 (10.00, 38.47)	<0.001	103.14±58.92	87.63±61.37	0.179	37.89 (10.00, 85.77)	17.96 (10.00, 60.33)	0.331
Sodium (mmol/L)	134.05±3.67	135.75±2.95	0.004	–	–		134.90±3.39	135.42±3.19	0.406	–	–	
ALT (U/L)	84.00 (33.50, 134.00)	44.50 (19.25, 109.75)	0.141	33.00 (23.00, 54.50)	25.00 (18.00, 37.00)	0.018	75.00 (25.00, 119.50)	52.00 (19.25, 116.75)	0.838	26.00 (18.50, 54.50)	27.00 (18.00, 43.00)	0.457
AST (U/L)	46.00 (27.00, 83.50)	36.00 (26.00, 55.75)	0.146	40.00 (29.50, 55.00)	38.00 (30.00, 48.00)	0.455	38.00 (28.00, 72.50)	38.00 (26.00, 61.00)	0.415	40.00 (29.00, 55.00)	38.00 (30.00, 46.00)	0.243
Total bilirubin (umol/L)	8.50 (4.10, 21.85)	6.19 (3.43, 9.85)	0.016	5.65 (3.35, 9.60)	5.00 (3.13, 7.58)	0.372	7.20 (4.97, 15.10)	6.40 (3.43, 12.35)	0.391	5.10 (3.65, 9.80)	5.10 (3.10, 8.10)	0.529
Albumin (g/L)	32.20±5.25	35.48±5.17	0.001	31.26±6.95	35.25±6.30	0.001	33.51±5.65	34.95±5.26	0.156	34.26±7.72	34.15±6.37	0.934
B/A ratio	0.35 (0.14, 0.74)	0.18 (0.09, 0.30)	0.004	0.21 (0.08, 0.32)	0.13 (0.09, 0.23)	0.127	0.18 (0.14, 0.53)	0.20 (0.09, 0.36)	0.280	0.15 (0.09, 0.25)	0.13 (0.08, 0.24)	0.456
CLI	3.11 (1.70, 4.70)	2.04 (1.04, 4.22)	0.061	2.33 (0.47, 4.98)	0.47 (0.26, 1.17)	<0.001	3.04 (1.58, 4.90)	2.19 (1.06, 4.19)	0.076	1.13 (0.23, 2.62)	0.52 (0.29, 1.83)	0.322
PNI	46.27±15.66	54.13±14.28	0.004	50.51±12.56	59.28±22.90	0.021	50.14±14.27	52.62±15.27	0.383	56.46±14.08	57.08±22.78	0.876
SII	1390.89 (573.39, 2418.69)	991.67 (593.93, 1876.36)	0.185	811.25 (384.46, 1840.79)	374.16 (197.26, 681.75)	<0.001	1287.49 (582.26, 2322.45)	992.99 (593.93, 1852.46)	0.328	489.83 (262.34, 953.43)	427.63 (216.25, 820.52)	0.621

Data are expressed as mean with standard deviation and median (interquartile range); IVIG, intravenous immunoglobulin; CAA, coronary artery aneurysm; NCAA, no coronary artery aneurysm; WBC, white blood cell count; NLR, neutrophil-to-lymphocyte count ratio; PLR, platelet-to-lymphocyte count ratio; CRP, C-reactive protein; ALT, alanine aminotransferase; AST, aspartate aminotransferase; B/A, total bilirubin-to-albumin; CLI, capillary leakage index; PNI, prognostic nutritional index; SII, systemic immune-inflammation index

### Results of the multi-factor logistic analysis

To determine the relative effect of each risk factor for IVIG resistance in KD, we performed logistic regression analysis. Before IVIG, the B/A ratio was included in the multivariable analysis instead of the two separate indicators. After IVIG, the CLI, calculated as the CRP level divided by the albumin level, was included in the multivariable analysis instead of the two separate indicators. The WBC and neutrophil counts, NLR, and PLR were not included in the multivariate logistic regression analysis because of their strong correlations with SII and PNI. Ultimately, the NLR, B/A ratio, PNI, and serum sodium level before IVIG, and the ALT level, PNI, SII, CLI, and serum hemoglobin level after IVIG were entered into the multivariable logistic regression analysis. All variables were tested for collinearity; however, no collinearity was present. The B/A ratio before IVIG and CLI and SII after IVIG were found to be significant independent predictors of IVIG resistance after adjusting for age, sex, and fever duration before admission (Table 3).

**Table 3:** Multivariate logistic analysis for predictors of IVIG resistance

Characteristics	Univariable		Multivariable		Adjust^#^		VIF
	odds ratio (95%CI)	*P*–value	odds ratio (95%CI)	*P*–value	odds ratio (95%CI)	*P*–value	
NLR (before IVIG)	1.121 (1.034–1.216)	0.006	0.923 (0.789–1.079)	0.313	0.930 (0.787–1.099)	0.395	2.008
Sodium (before IVIG)	0.850 (0.757–0.954)	0.006	0.965 (0.838–1.111)	0.618	0.970 (0.840–1.119)	0.675	1.189
B/A ratio (before IVIG)	3.799 (1.622–8.895)	0.002	2.705 (1.036–7.063)	0.042	2.716 (1.023–7.211)	0.045	1.429
PNI (before IVIG)	0.949 (0.916–0.983)	0.004	0.975 (0.932–1.020)	0.279	0.960 (0.923–1.016)	0.192	1.502
Hemoglobin (after IVIG)	0.955 (0.928–0.983)	0.002	0.961 (0.925–0.998)	0.038	0.963 (0.926–1.002)	0.060	1.132
ALT (after IVIG)	1.010 (1.000–1.019)	0.041	1.011 (0.999–1.022)	0.066	1.011 (1.000–1.023)	0.056	1.099
CLI (after IVIG)	1.501 (1.248–1.805)	<0.001	1.421 (1.139–1.773)	0.002	1.415 (1.131–1.769)	0.002	1.251
PNI (after IVIG)	0.949 (0.916–0.983)	0.003	1.002 (0.972–1.034)	0.876	1.004 (0.974–1.034)	0.819	1.205
SII (after IVIG)	1.001 (1.000–1.002)	<0.001	1.001 (1.000–1.001)	0.012	1.001 (1.000–1.001)	0.011	1.280

^#^indicates a significant relationship after correction for age and fever duration before admission; IVIG, intravenous immunoglobulin; CI, confidence interval; VIF, variance inflation factors; NLR, neutrophil-to-lymphocyte count ratio; B/A, total bilirubin-to-albumin; PNI, prognostic nutritional index; ALT, alanine aminotransferase; CLI, capillary leakage index; SII, systemic immune-inflammation index

### Fluctuations of risk factors pre- and post-treatment

**Fig. 2:**
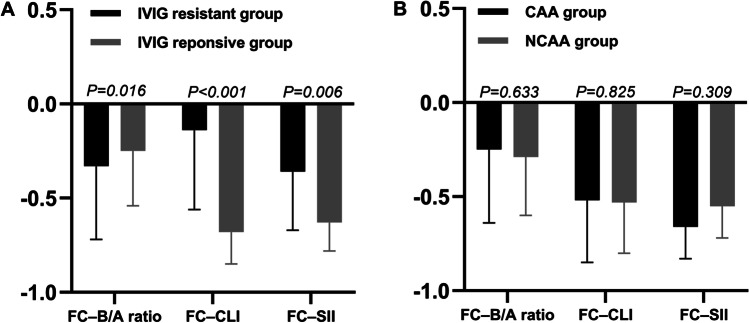
Comparisons of fractional change in risk factors between subgroups before and after treatment. IVIG, intravenous immunoglobulin; CAA, coronary artery aneurysm; NCAA, no coronary artery aneurysm; FC, fractional change = ([data at 24 h to 36 h after IVIG]−[data before IVIG])/data before IVIG; B/A, total bilirubin-to-albumin; CLI, capillary leakage index; SII, systemic immune-inflammation index

We selected the following variables that showed significant differences between the IVIG-resistant and IVIG-responsive groups after multivariable logistic regression analysis: B/A ratio, CLI, and SII. Before and after IVIG, the significant variables were compared between subgroups for FC (see Supplementary File 1); there were a larger decline in the B/A ratio and smaller decline in CLI and SII pre- and post-treatment in the IVIG-resistant group than in the IVIG-responsive group (Fig. 2A). However, the FC was not statistically different between the patients with and without CAA (Fig. 2B).

### Predictive value for IVIG resistance

**Fig. 3:**
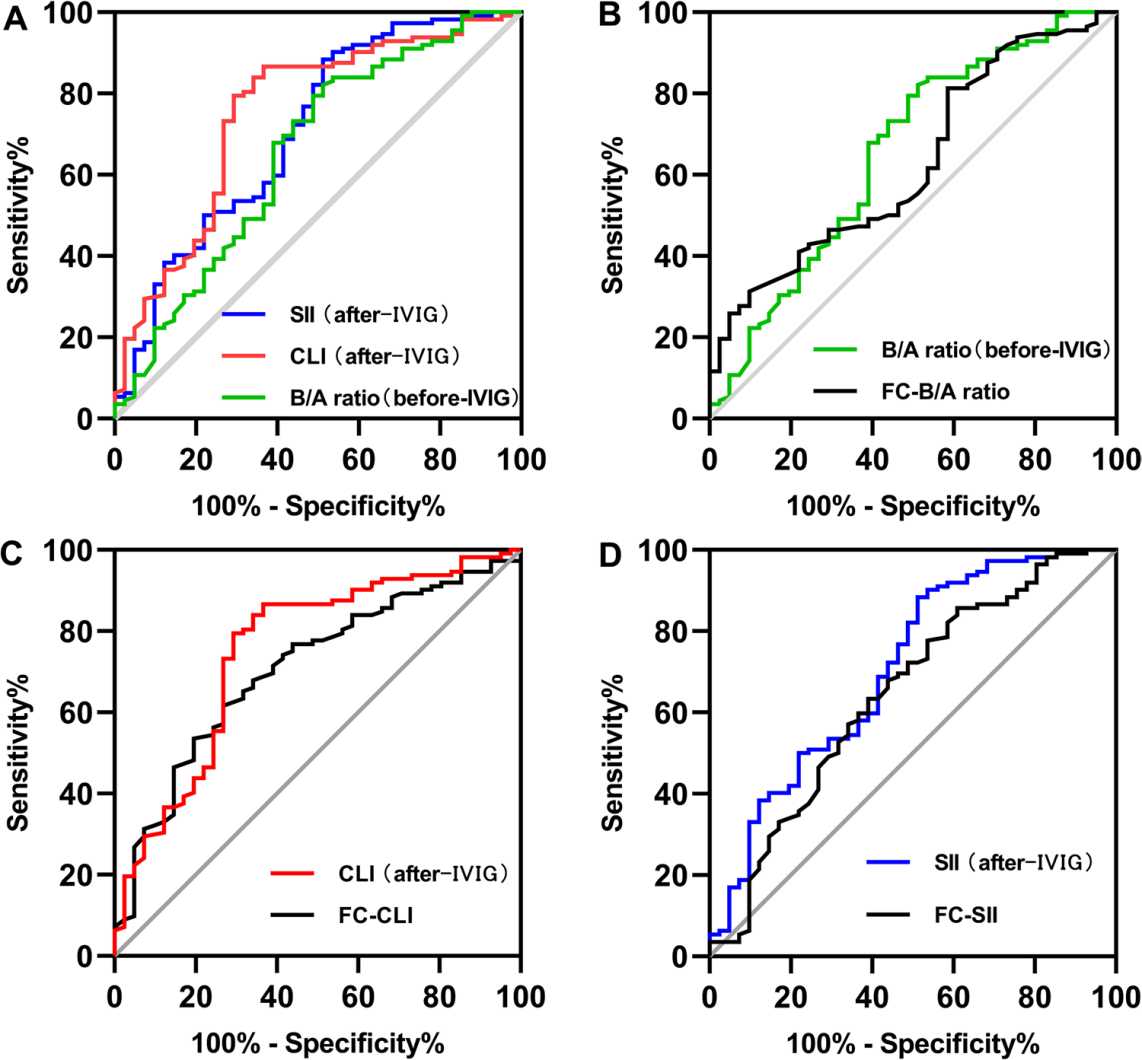
Receiver operating characteristic curves for the risk factors as well as its fractional changes in IVIG-resistant patients with Kawasaki disease. IVIG, intravenous immunoglobulin; FC, fractional change = ([data at 24 h to 36 h after IVIG]−[data before IVIG])/data before IVIG; B/A, total bilirubin-to-albumin; CLI, capillary leakage index; SII, systemic immune-inflammation index

The cutoff values of the parameters were determined using ROC curves and are shown in Supplementary File 1. To assess the differential contributions of the various risk factors to the prediction of IVIG resistance, we calculated the ROC curves for both the risk factors and their fluctuations pre- and post-treatment. The cutoff value of the B/A ratio before IVIG of 0.363 was 49% sensitive and 82% specific (AUC = 0.654; 95% CI, 0.549–0.758, *p* = 0.004). Meanwhile, the cutoff value of CLI after IVIG of 1.49 was 71% sensitive and 80% specific (AUC = 0.751; 95% CI, 0.659–0.843, *p* < 0.001). The cutoff value of SII after IVIG of 1,006.11 was 49% sensitive and 88% specific (AUC=0.706; 95% CI, 0.608–0.805, *p* < 0.001). Interestingly, in terms of predictive ability, CLI and SII after IVIG were superior to the B/A ratio before IVIG (Fig. 3A); however, the predictive value of the FC in these risk factors was not superior to that of raw data (Figs. 3B, C, D).

## Discussion

Due to the lack of consensus for any single risk factor, parameters merging the risk factors have become a research hotspot for the prediction of IVIG resistance in patients with KD as these indices may be more reliable than individual parameters. In the present study, the incidence of IVIG resistance was 26.8%, which was similar to previous reports ranging from 7.8% to 38.3% [5, 7, 8, 23–29]. Our study identified that the B/A ratio before IVIG and CLI and SII after IVIG were significantly and positively associated with IVIG resistance, and IVIG-resistant patients had a larger decline in the B/A ratio and smaller decline in CLI and SII pre- and post-treatment than IVIG-responsive patients; however, no significant differences in the parameters and their fluctuations pre- and post-treatment were found between patients with and without CAA. This suggests that different pathologies could be responsible for IVIG resistance and CAA development and are not merely associated with systemic inflammation, which agrees with previous studies [30–32]. Additionally, the post-treatment parameters were superior to the pre-treatment parameters for the prediction of IVIG resistance; however, the predictive ability of fluctuations in risk factors pre- and post-treatment were not superior to that of baseline values. This study is the first to simultaneously evaluate the clinical and laboratory characteristics and their fluctuations pre- and post-treatment in patients with KD and its subgroups. Further investigations are warranted to better elucidate the pathophysiology of KD as we continue to expand our understanding of IVIG resistance and CAA development.

In our previous work, we found that the B/A ratio was highly associated with IVIG resistance in high-risk patients, with a Xie Liping risk score of ≥5 points; the same results were observed in high-risk patients with KD as defined by a Kobayashi score of ≥4 points [33]. Based on the findings of previous studies [34–36], hepatic dysfunction is a common complication during acute KD episodes and is characterized by elevated serum liver enzymes, hypoalbuminemia, and hyperbilirubinemia. The underlying mechanism of hepatic dysfunction in patients with KD remains unclear and could be associated with infectious agents, inflammatory mediators, or a combination of both. Hyperbilirubinemia, which is caused by the lysis of hepatocytes and is indicative of intense inflammatory responses, and hypoalbuminemia are more representative of increased capillary permeability associated with systemic vasculitis that leads to a higher leakage of serum albumin [37, 38]. A single inflammatory parameter may be easily influenced by other factors; therefore, merging information of various inflammatory parameters may theoretically be more reliable and have the potential to be a powerful candidate marker to evaluate the inflammatory status. As expected, our study found that the B/A ratio before treatment was a risk factor associated with IVIG resistance and that there was a larger decline in the fluctuations of B/A ratio pre- and post-treatment in IVIG-resistant patients than in IVIG-responsive patients. To the best of our knowledge, the present study is the first to suggest that a high value and large fluctuations in the B/A ratio are peculiarities in IVIG-resistant patients with KD and that a high B/A ratio before IVIG could be a supportive factor in the assessment of IVIG resistance risk in patients with KD; however, future prospective studies are needed to confirm this finding.

The CLI can reflect the degree of inflammation in numerous diseases such as acute coronary syndromes, sepsis, and tumors [39–43]. A study in 2020 found that the CLI can serve as a novel predictive marker for formation of coronary artery lesions and IVIG resistance in patients with KD in a Taiwanese population [44]. Thereafter, another study with a large sample size and prospective approach identified that the CLI was significantly higher in patients with IVIG resistance and was an independent risk factor for both initial and repeated IVIG resistance; however, its predictive value was not superior to that of CRP or albumin [45]. Unfortunately, our results do not agree with the conclusions of the two studies as our study found that IVIG-resistant patients had a higher CLI pre- and post-treatment than IVIG-responsive patients, but only differences in CLI after treatment were statistically significant. Additionally, a lower decrease in the fluctuations of CLI pre- and post-treatment was observed in IVIG-resistant patients than in IVIG-responsive patients. Multivariate analysis showed that the CLI after treatment was a risk factor for predicting IVIG resistance in patients with KD and that CLI outperformed other risk factors by having the largest AUC of ROC. This suggests that the laboratory data post-IVIG are more meaningful than the pre-IVIG data, which was corroborated in the present study and partially consistent with that of Sang et al. [46]. Unfortunately, the fluctuation of CLI pre- and post-treatment was not significantly better than the raw data in their predictive value in this study. After reviewing the literature, this study is the first to clarify the role of dynamic changes in CLI in children with KD, indicating the importance of monitoring the CLI before and after treatment as an indicator of IVIG resistance in patients with KD.

Immune system hyperactivation has been detected during the acute phase of KD in autopsy and animal models, suggesting that inflammatory biomarkers can help improve KD diagnosis and predict prognosis [47–49]. The WBC and its subtypes have become classic inflammatory markers; consequently, the number of peripheral leukocyte counts will be altered when the immune system of the host is stimulated by systemic inflammation. It is generally agreed that neutrophils are non-specific inflammatory markers, while lymphocytes are immunomodulatory markers; hence, the NLR reflects the balance between inflammatory reactions and immune responses, and the NLR elevation can be considered a marker of exacerbation of an inflammatory process [50, 51]. Consistent with previous findings [5, 21, 46, 52–54], our results showed that IVIG-resistant patients had a significantly higher NLR both pre- and post-treatment than IVIG-responsive patients. Notably, SII, which combines the NLR and platelet counts, was proposed as an independent risk factor for IVIG resistance and several cardiovascular complications in patients with KD; however, opposite findings were reported in subgroup analyses of patients with coronary artery lesions [19, 22]. In the present study, IVIG-resistant patients had a higher SII and a lower decrease in its fluctuations pre- and post-treatment than IVIG-responsive patients. Additionally, a high SII after treatment was found to be significantly associated with IVIG resistance but not CAA development, which is consistent with the results of Liu et al. [22] but not of Rumeysa et al. [19], possibly because of the different days of detection of illness onset. Interestingly, our study found that patients with KD had a lower PNI, which is an index for nutritional assessment, both in the IVIG-resistant and CAA groups when compared to their respective counterparts, as in several previous studies [15–19]. It is worth noting that the lowest PNI value in the IVIG-resistant group occurred on day 6, which corresponds to the highest SII value in the present study (Supplementary Fig.); this is in line with previous studies [18, 55–57] and suggests that the PNI or SII obtained on the sixth day from fever onset may possess good predictive power for IVIG resistance in patients with KD. However, we found that both the PNI and SII before treatment were not significantly associated with IVIG resistance and CAA development, which may be due to the small sample size. Thus, further research is warranted to verify these conflicting results regarding SII and PNI.

There were some limitations to the present study. First, selection bias may have occurred because the study was retrospective and performed in a single institution with a small number of patients. Furthermore, 10.5% (16 of 153) of the patients in our cohort were recurrent IVIG-resistant patients; however, the results obtained for IVIG-resistant patients without steroid therapy were the same (Supplementary File 2). Second, the absence of a gold standard for diagnosing KD might have erroneously classified subjects as we cannot rule out the possibility that at least some patients with incomplete KD in our study might not have had KD but another illness closely resembling KD. This is especially relevant during the COVID-19 pandemic as multisystem inflammatory syndrome in children, which has overlapping clinical features with KD, has been reported. However, SARS-CoV-2 nucleic acid testing was negative in all patients before they were admitted as required by the relevant epidemic prevention and control guidelines. We hopefully minimized this possibility by carefully excluding patients with a more plausible cause of other infectious diseases. Lastly, because of non-routine testing, some risk factors for IVIG resistance that have been reported were not considered in the study design, including levels of serum interleukin-6, brain natriuretic peptide, and immune function parameters. Hence, further well-designed prospective studies on a larger scale may be needed.

## Conclusions

The B/A ratio before IVIG and CLI and SII after IVIG were risk factors for IVIG resistance in patients with KD, independent of CAA development. Furthermore, the post-treatment parameters were superior to the pre-treatment parameters in terms of their predictive ability. Therefore, rapid and repeated assessment of risk factors before and after IVIG treatment may be useful for identifying patients with KD who have a high risk of IVIG resistance to guide further therapy strategies.

## Supplementary Information

Supplementary File 1 (DOCX 20 kb)

Supplementary File 2 (DOCX 17 kb)

Supplementary Figure (PNG 161 kb)

High Resolution Image (TIF 1564 kb)

## Data Availability

The raw data supporting the conclusions of this article will be made available by the authors, without undue reservation.
