# Supplementary material for: Evaluation of laboratory predictors for intravenous immunoglobulin resistance and coronary artery aneurysm in Kawasaki Disease before and after therapy
Source: Clin Rheumatol. 2022 Sep 21;42(1):167–77. doi: 10.1007/s10067-022-06366-x (PMC9491265; doi:10.1007/s10067-022-06366-x)
Supplement: Supplementary file 1 — (DOCX 20 kb) [file 10067_2022_6366_MOESM1_ESM.docx]

**Receiver-operating characteristic curves indicating comparisons of discriminative ability for risk factors**

We selected the following variables that showed significant differences between the intravenous immunoglobulin (IVIG)-resistant and IVIG-responsive groups after multivariable logistic regression analysis: total bilirubin-to-albumin (B/A) ratio before IVIG, capillary leakage index (CLI) and systemic immune-inflammation index (SII) after IVIG, and the significant variables were compared between subgroups for fractional change (FC, defined as FC=([data at 24 h to 36 h after IVIG]−[data before IVIG])/data before IVIG). Patients with IVIG-resistant had a larger change in the FC–B/A ratio but smaller changes in the FC–CLI and FC–SII than those in patients with IVIG-responsive; however, the FC was not statistically different between the patients with and without CAA (Table 1). The cutoff value of the three parameters was determined by receiver operating characteristic (ROC) curves: the B/A ratio before IVIG cutoff value of 0.363 provided a sensitivity of 49% and a specificity of 82%, with an area under the curve (AUC) of 0.654 (95% CI, 0.549-0.758, *p*=0.004); the CLI after IVIG cutoff value of 1.49 provided a 71% sensitivity and a 80% specificity (AUC=0.751 [95% CI, 0.659-0.843, *p*<0.001]); and the SII after IVIG cutoff value of 1006.11 yielded 49% sensitivity and 88% specificity (AUC=0.706 [95% CI, 0.608-0.805, *p*<0.001]). In order to assess the differential contribution of the various risk factors to the prediction of IVIG resistance, we next calculated the ROC curves for both risk factors as well as its fractional changes. Interestingly, it should be noted that the post-treatment parameters were superior to the pre-treatment parameters in the predictive role, however, the predictive value of the FC in these risk factors was not superior to that of raw data (Table 2).

Table 1 Fractional change of laboratory parameters, by subgroups

|  | Fractional change (FC) | | *P*–*value* | Fractional change (FC) | | *P*–*value* |
| --- | --- | --- | --- | --- | --- | --- |
|  | IVIG-resistant | IVIG-responsive |  | CAA | NCAA |  |
| B/A ratio | ﹣0.33  (﹣0.72, -0.10) | ﹣0.25  (-0.54, -0.14) | 0.016 | ﹣0.25  (﹣0.64, 0.04) | ﹣0.29  (-0.60, 0.02) | 0.633 |
| CLI | ﹣0.14  (﹣0.56, -0.31) | ﹣0.68  (-0.85, -0.27) | ＜0.001 | ﹣0.52  (﹣0.85, -0.05) | ﹣0.53  (-0.80, -0.08) | 0.825 |
| SII | ﹣0.36  (﹣0.67, 0.26) | ﹣0.63  (-0.78, -0.26) | 0.006 | ﹣0.66  (﹣0.83, -0.11) | ﹣0.55  (-0.72, -0.18) | 0.309 |

FC, fractional change=([data at 24 h to 36 h after IVIG]−[data before IVIG])/data before IVIG; IVIG, intravenous immunoglobulin; CAA, coronary artery aneurysm; NCAA, no coronary artery aneurysm; B/A, total bilirubin-to-albumin; CLI, capillary leakage index; SII, systemic immune-inflammation index.

Table 2 Comparisons of risk factors as well as its fractional changes for IVIG resistance

| Risk factors | AUC | *P*–value | 95%CI | cutoff value | sensitivity (%) | specificity (%) |
| --- | --- | --- | --- | --- | --- | --- |
| B/A ratio (before IVIG) | 0.654 | 0.004 | 0.549–0.758 | 0.363 | 49 | 82 |
| CLI (after IVIG) | 0.751 | ＜0.001 | 0.659–0.843 | 1.49 | 71 | 80 |
| SII (after IVIG) | 0.706 | ＜0.001 | 0.608–0.805 | 1006.11 | 49 | 88 |
| FC–B/A ratio | 0.627 | 0.016 | 0.529–0.725 | - | - | - |
| FC–CLI | 0.706 | ＜0.001 | 0.616–0.796 | - | - | - |
| FC–SII | 0.646 | 0.006 | 0.542–0.750 | - | - | - |

IVIG, intravenous immunoglobulin; AUC area under the curve; CI, confidence interval; B/A, total bilirubin-to-albumin; CLI, capillary leakage index; SII, systemic immune-inflammation index; FC, fractional change=([data at 24 h to 36 h after IVIG]−[data before IVIG])/data before IVIG.
