# Supplementary material for: Evaluation of laboratory predictors for intravenous immunoglobulin resistance and coronary artery aneurysm in Kawasaki Disease before and after therapy
Source: Clin Rheumatol. 2022 Sep 21;42(1):167–77. doi: 10.1007/s10067-022-06366-x (PMC9491265; doi:10.1007/s10067-022-06366-x)
Supplement: Supplementary file 2 — (DOCX 17 kb) [file 10067_2022_6366_MOESM2_ESM.docx]

**Results of the multi-factor logistic analysis for intravenous immunoglobulin resistance**

A total of 153 children were include in this study, 16 (10.5%) had received steroid therapy in addition to the second dose of gammaglobulin therapy were excluded. Of the remaining 137 patients, 25 (18.2%) had initial intravenous immunoglobulin (IVIG) resistance and 30 (21.9%) with coronary artery aneurysm (CAA) development. The mean age of IVIG-resistant KD was 26 months with interquartile range (IQR) of 13–51.5 months, and with a male-to-female ratio of 2.6:1 (18 boys and 7 girls). The mean age of IVIG-responsive KD was 24 months with IQR of 14.3–40.5 months, and with a male-to-female ratio of 2.5:1 (80 boys and 32 girls). Univariate analysis showed that the total bilirubin-to-albumin (B/A) ratio before IVIG, and capillary leakage index (CLI) and systemic immune-inflammation index (SII) after IVIG significantly differ between the two groups, and multivariate logistic regression analysis suggested that the B/A ratio before IVIG, and CLI and SII after IVIG were significantly associated with IVIG resistance after adjusting for age, sex and fever duration before admission (Table 1).

Table 1 Multivariate logistic analysis for predictors of IVIG resistance

| characteristic | Univariable | | Multivariable | | Adjust^#^ | |
| --- | --- | --- | --- | --- | --- | --- |
|  | odds ratio (95%CI) | *P*value | odds ratio (95%CI) | *P*value | odds ratio (95%CI) | *P*value |
| B/A ratio (before IVIG) | 3.768 (1.507–9.424) | 0.005 | 3.061 (1.297–7.225) | 0.011 | 3.109 (1.274–7.586) | 0.013 |
| CLI (after IVIG) | 1.437 (1.169–1.766) | 0.001 | 1.396 (1.123–1.734) | 0.003 | 1.411 (1.131–1.761) | 0.002 |
| SII (after IVIG) | 1.001 (1.000–1.001) | 0.009 | 1.001 (1.000–1.001) | 0.053 | 1.001 (1.000–1.001) | 0.040 |

^#^indicates a significant relationship after correction for age, sex and fever duration before admission; IVIG, intravenous immunoglobulin; CI, confidence interval; B/A, total bilirubin-to-albumin; CLI, capillary leakage index; SII, systemic immune-inflammation index.
