# Supplementary figures and images for: Evaluation of laboratory predictors for intravenous immunoglobulin resistance and coronary artery aneurysm in Kawasaki Disease before and after therapy
Source: Clin Rheumatol. 2022 Sep 21;42(1):167–77. doi: 10.1007/s10067-022-06366-x (PMC9491265; doi:10.1007/s10067-022-06366-x)

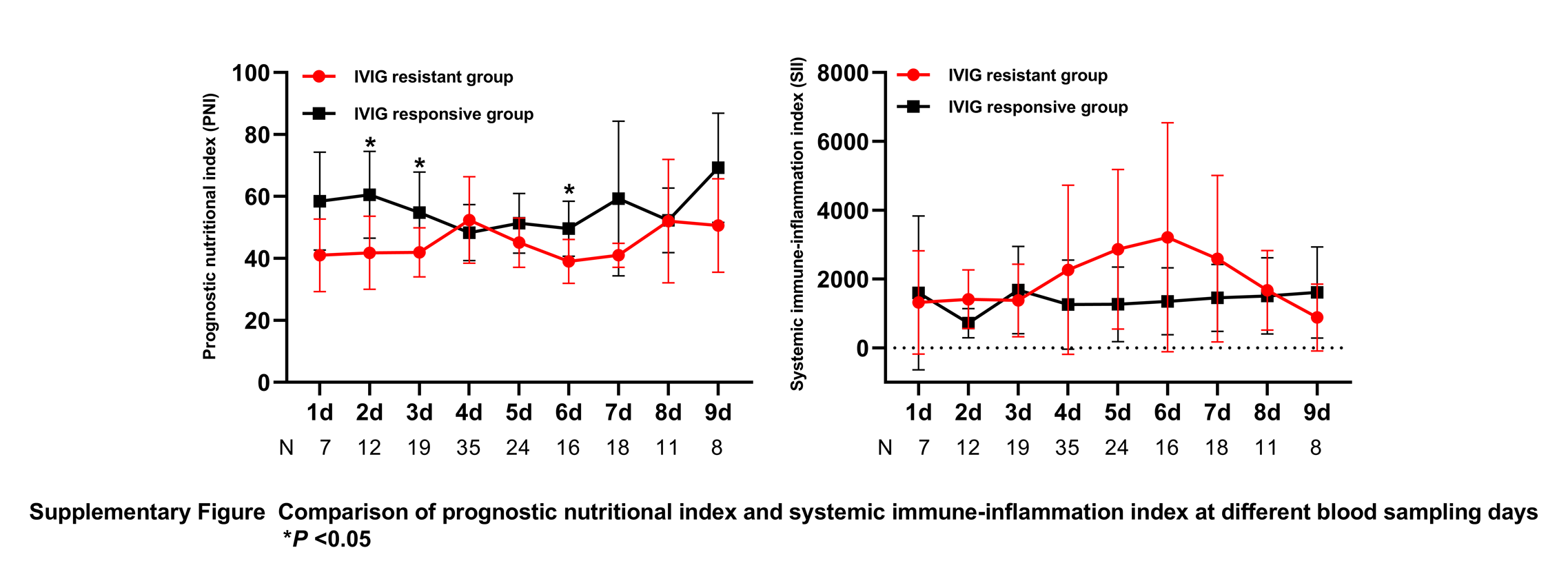

Supplement: Supplementary file 3 — (PNG 161 kb) [file 10067_2022_6366_Fig4_ESM.png]

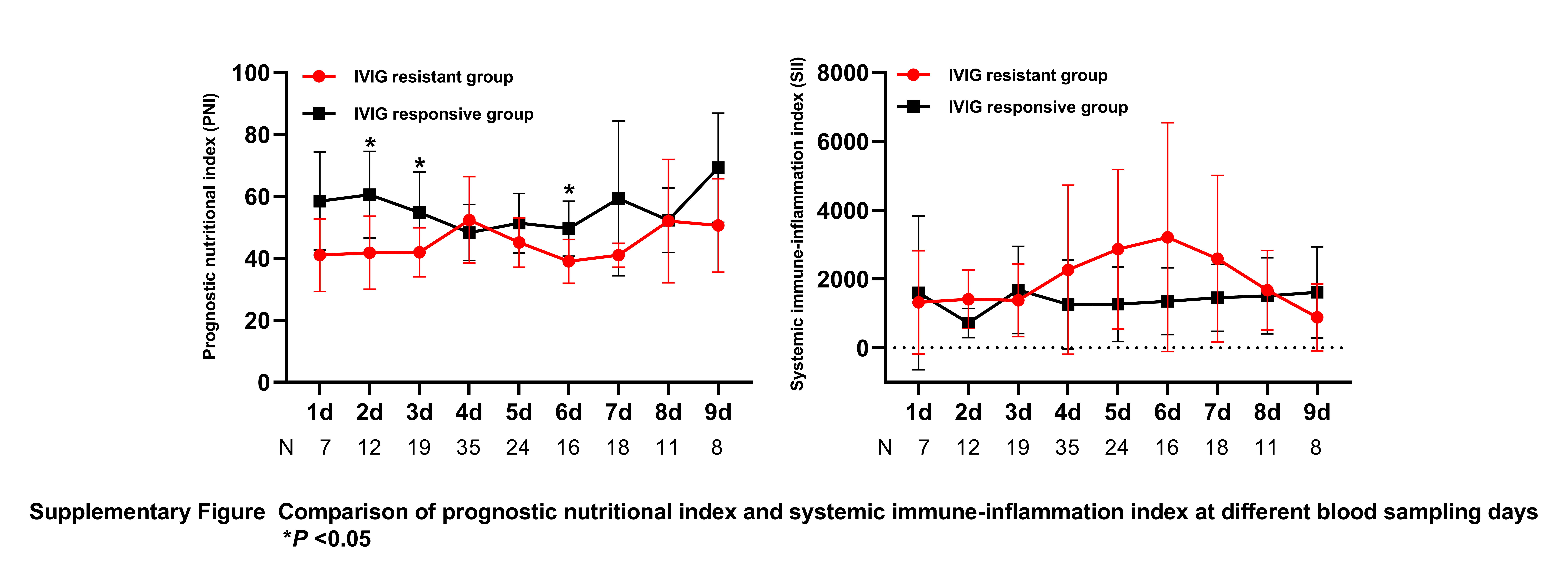

Supplement: Supplementary file 4 — High Resolution Image (TIF 1564 kb) [file 10067_2022_6366_MOESM3_ESM.tif]
